# An Editorial on the Special Issue “Genetic Basis of Human Diseases”

**DOI:** 10.3390/life14060691

**Published:** 2024-05-28

**Authors:** Mikhail Churnosov

**Affiliations:** Department of Medical Biological Disciplines, Belgorod State National Research University, 308015 Belgorod, Russia; churnosov@bsu.edu.ru

The role of heredity in the emergence of the overwhelming majority of human diseases is currently considered proven and has significant importance [1,2,3]. The contribution of hereditary factors in the occurrence of a significant number of human diseases such as osteoarthritis (50%) [1], uterine leiomyoma (55–69%) [2], breast cancer (31–41%) [3], endometriosis (47–51%) [2] and others is notable and may exceed 50% [2]. Based on this, the search for specific genetic factors (candidate genes/polymorphisms) that form the “basis” of the heredity of human diseases is of great scientific (fundamental) and practical (applied) importance. This is because it will not only allow us to better understand the diseases’ pathogenesis (genetic causes/mechanisms of their development) but also create prerequisites for a wider implementation of genetic approaches (methods, technologies) in clinical medicine.

The scientific papers presented in this Special Issue, titled “Genetic Basis of Human Diseases”, are aimed at finding genetic factors that determine the risk of developing such common and socially significant human diseases, such as uterine leiomyoma, knee osteoarthritis, Alzheimer’s disease, obesity, and psoriasis.

In a review article by Fedotova et al. [4], new data on the role of the hypoxia-induced factor (HIF) in the development of uterine leiomyoma are presented. Summarizing the current literature on this issue, the authors provide convincing pathogenetic justifications (link with proliferation, apoptosis, metabolism, and regulation of cellular oxygen/nutrients availability) for the association of the HIF family’s (involved in hypoxic metabolism) genes/proteins with the development of uterine leiomyoma.

In a replicative genetic study of knee osteoarthritis, Novakov et al. [5] showed that polymorphisms—which are associated with the risk of developing the disease, according to previously performed genome-wide studies—correlate with the occurrence of the disorder in residents of Central Russia only with certain intergenic interactions (twelve models of genetic interactions in eight out of the ten studied loci were modeled in the study). As the authors have shown, the risk effects of these polymorphisms in relation to knee osteoarthritis largely depend on modifying factors, such as sex and BMI [6,7].

Ocenasova et al.’s study was devoted to the assessment of polymorphism of matrix metalloproteinase genes’ involvement in the predisposition to Alzheimer’s disease in the Slovak population [8]. The authors found an association with the age of onset of the disease of polymorphism rs243866 of the matrix metalloproteinase 2 gene, and the GG genotype at this locus marked an earlier manifestation of the pathology. This work once again convincingly demonstrates the important medical significance of the genetic variant of matrix metalloproteinases, which has been repeatedly demonstrated in previously performed works [9,10]. Moreover, it indicates the key importance of matrix metalloproteinase genes in the human organism, both in normal and pathological conditions.

Interesting data on the relationship of polymorphic variants of the *TCN-2* gene (776C>G) with vitamin B12 levels (depending on BMI) were obtained in the work of Ashfaq et al. [11]. The authors showed that, on the one hand, polymorphism of the *TCN-2* gene (776C>G) affects one’s predisposition to the development of obesity. On the other hand, obesity and overweight correlate with low levels of vitamin B12, which is an important modifier of the body’s lipid profile. The data obtained by the authors once again indicate the importance of gene–environment interactions in the occurrence of pathology. Additionally, they are consistent with the other data within the literature on the primary importance of obesity in “genotype–environment” interactions in the development of various diseases [6,10,12].

Efanova et al.’s work [13] proved, for the first time, the connection of *GCLC* gene’s polymorphism (which is involved in glutathione metabolism) with the development of a widespread skin disease, such as psoriasis. The authors demonstrated significant sex-specific features of the association of the *GCLC* gene’s genetic variant with the disease, the modifying effect of smoking/alcohol consumption on these associations and established significant correlations of the considered genetic determinants with various clinical signs, such as the disorder’s age of onset, the psoriatic triad and the nature of the target organ’s lesion—the skin.

In conclusion, I would like to note that despite the significant contributions made to our understanding of the pathogenesis of a number of human diseases (uterine leiomyoma, knee osteoarthritis, Alzheimer’s disease, obesity, and psoriasis) by the works presented in this Special Issue (“Genetic Basis of Human Diseases”), further research in this area is certainly needed. Carrying out more studies would allow the scientific community to advance our understanding of the genetic basis of these diseases’ occurrences and would enable us to use this knowledge in clinical practice.
